# Characterization and retinal neuron differentiation of WERI-Rb1 cancer stem cells

**Published:** 2012-09-24

**Authors:** Huiling Hu, Fei Deng, Ying Liu, Mengfei Chen, Xiulan Zhang, Xuerong Sun, Zhizhang Dong, Xiaohong Liu, Jian Ge

**Affiliations:** State Key Laboratory of Ophthalmology, Zhongshan Ophthalmic Center, Sun Yat-sen University, Guangzhou, China

## Abstract

**Purpose:**

The evidence is increasing that cancer stem cells (CSCs) expressing embryonic and neuronal stem cell markers are present in human retinoblastoma (Rb). This study was conducted to determine whether stem-like cancer cells (SLCCs) in Rb express retinal stem cell–related genes and whether SLCCs can directly differentiate into retinal neurons.

**Methods:**

The cancer stem cell characteristics in WERI-Rb1 cells were determined with Hoechst 33,342 staining, clone formation assay, and CD133 flow cytometry. The expression of embryonic stem cell and retinal stem cell–related genes was analyzed with real-time PCR and immunofluorescence. The SLCCs were induced to differentiate into retinal neurons by the addition of Dickkopf-related protein 1 and Lefty-A.

**Results:**

A small but persistent population of cells excluding Hoechst dye in a verapamil-sensitive manner exhibited a cancer stem cell–like phenotype. The SLCCs displayed highly clonogenic abilities and increased CD133 expression with isolation and expansion in culture in serum-free medium. By comparing the expression of stem cell markers, we found Oct3/4 was more highly expressed in the SLCCs than in human embryonic stem cells. Together with the properties of intrinsic retinal stem cell–related gene expression, we found SLCCs can be induced into neuron-like cells that express glial fibrillary acidic protein and rhodopsin (a photoreceptor cell marker).

**Conclusions:**

These findings provide new insight into cancer stem cells and used a strategy of an artificial change of cancer stem cell fate with transcription factors.

## Introduction

Stem cells are defined as cells that can perpetuate themselves through self-renewal and generate mature cells of a particular tissue through differentiation. There are two broad types of stem cells: embryonic and adult stem cells [1]. Recently, focus has been increasing on a specific type of stem cell—cancer stem cells (CSCs). CSCs are cells found within tumors or hematological cancers and share characteristics of normal stem cells, such as extensive proliferation, self-renewal, and multipotency [1]. CSCs were first found in acute myeloid leukemia [1,2] and were later found in several cancers, including breast cancer [3], human brain tumors [4], and ovarian cancer [5].

Side population (SP) cells are a particular cell population enriched in primitive and undifferentiated cells [6-8]. Analysis of the hematopoietic system has shown that bone marrow stem cells contain a subpopulation that effluxes the DNA binding dye, Hoechst 33,342, out of the cell membrane. These cells are called SP cells and are shown to have stem cell characteristics and enrich the stem cell population [9]. SP cells isolated from tumors have proved to be an attractive alternative for studying CSCs [10].

Retinoblastoma (Rb), the most common childhood tumor of the eye, is caused by the inactivation of both alleles of the retinoblastoma 1 (*Rb1*) gene [11]. Previous reports have demonstrated that stem cells expressing embryonic and neuronal stem cell markers are present in human Rb [12-14]. In 2007, our research group successfully designed culture methods for selecting and expanding stem-like cancer cells (SLCCs) in Rb [15]. Interestingly, the cultured cells also expressed retinal development–related genes.

Based on these characteristics of SLCCs in Rb, we asked whether SLCCs in WERI-Rb1 express embryonic and retinal stem cell–related genes and whether the cells can differentiate directly into retinal neurons, as human embryonic stem cells (hESCs) [16-18] and induced pluripotent stem cells (iPSCs) [19] can.

## Methods

### Cell culture

The human retinoblastoma cell line, WERI-Rb1, obtained from the American Type Culture Collection (ATCC, Manassas, VA), was maintained in RPMI-1640 Medium (Hyclone, Logan, UT) with 10% fetal bovine serum (FBS; Gibco, Carlsbad, CA) [20]. We used a serum-free medium (SFM) to isolate and maintain the SLCCs from WERI-Rb1 according to described procedures [15,21,22]. Briefly, WERI-Rb1 cells were seeded in SFM for SLCCs selection. Ten to 14 days later, large neurosphere-like tumor spheres were harvested and passaged by 1 to 2 or 1 to 3 in SFM. The SFM contained 20 ng/ml basic fibroblast growth factor (bFGF; PeproTech, Rocky Hill, NJ), 20 ng/ml epidermal growth factor (EGF; PeproTech), and 20 ng/ml leukemia inhibitory factor (LIF; Millipore, Temecula, CA) at a density of 100,000 cells/ml [15,21,23]. Neurosphere-like tumor spheres were passaged with mechanical dissociation to single cells using a Pasteur pipette every 3–4 days and reseeded in the medium described above at 100,000 cells/ml. All of the cells used in this study were placed in a 37 °C humidified incubator with 5% CO_2_ and observed under inverted microscopy every other day.

The hESCs line BG01V was obtained from the ATCC and propagated on mitomycin-C-inactivated mouse embryonic fibroblasts (MEFs; Millipore) in hESC medium: Dulbecco's Modified Eagle Medium/F12 (DMEM/F12 ; Gibco; 1:1) with 20% knockout serum replacement, 0.1 mM nonessential amino acids, 2 mM L-glutamine, 50 units/ml penicillin G, 50 μg/ml streptomycin sulfate (all from Gibco), 0.1 mM beta-mercaptoethanol (Millipore), and 4 ng/ml bFGF (PeproTech). The hESCs were passaged enzymatically every 7 days. Briefly, cells were washed with DMEM/F12 (1:1) and then incubated with collagenase IV (1 mg/ml in DMEM/F12, Gibco) for 40 min at 37 °C and 5% CO_2_. Any semiadhered hESC colonies were pushed off the plate with a plastic pipette, and colonies were sedimented via centrifugation. Cell colonies were broken up into smaller cell aggregates by trituration of the harvested colonies in hESC medium. Triturated cells were then plated on new mitomycin-C-inactivated MEFs and incubated at 37 °C with 5% CO_2_ for 48 h, and media were changed daily.

### Hoechst staining for side population detection

**Figure 1:**
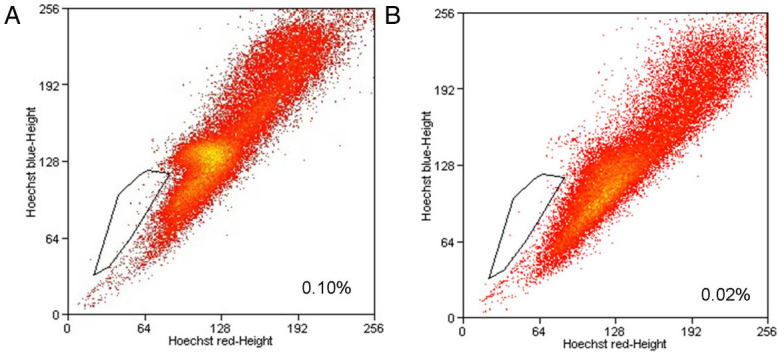
Existence of stem-like side population cells in the human retinoblastoma WERI-Rb1 cell line. The cells were stained with Hoechst 33,342 and sorted with flow cytometry. **A**: A small population of cells (0.075±0.017%) was identified. **B**: Addition of 50 μM verapamil markedly reduced the staining of the SP cells. (n=3; x-axis, Hoechst red fluorescence intensity; y-axis, Hoechst blue fluorescence intensity).

The Hoechst 33,342 labeling and flow cytometry analysis of the SP cells were performed following the Geng et al. [24,] procedure with minor modifications. Single suspension WERI-Rb1 cells were stained with Hoechst 33,342 dye for 90 min. Because Hoechst 33,342 extrudes from cells treated with verapamil, a subset of the cells were incubated with verapamil to determine whether this would block the fluorescent efflux of SP cells. The cells were analyzed with fluorescence-activated cell sorting when they had reached a logarithmic growth phase (24 h after replating). The cells were resuspended at 10^6^/ml in RPMI-1640 supplemented with 2% FBS and preincubated in a 1.5-ml Eppendorf tube at 37 °C for 10 min. The cells were then labeled in the same medium at 37 °C for 90 min with 5 μg/ml Hoechst 33,342 (Sigma-Aldrich, St. Louis, MO), a DNA-binding dye, either alone or combined with 50 μM verapamil (Sigma-Aldrich), an inhibitor of some adenosine-5′-triphosphate-binding cassette transporters. The cells were washed twice with cold PBS buffer (containing 137 mM sodium chloride, 2.7 mM potassium chloride, 4.3 mM disodium hydrogen phosphate and 1.4 mM potassium dihydrogen phosphate, PH 7.4; Gibco) containing 2% FBS, and flow cytometry analysis was performed using a MoFlo XDP cell sorter (Beckman Coulter, Fullerton, CA). The Hoechst 33,342 dye was excited with a ultraviolet laser at 350 nm, and the fluorescence emission was detected through 450/40-nm band-pass (Hoechst blue) and 695/40-nm long filters (Hoechst red), respectively. The SP, non-SP, and unfractionated or total population cells were gated as shown in Figure 1.

### Clone formation assay

The cells in suspension were carefully counted and prepared at 10^5^/ml in culture medium using a cytometer. The cells were then serially diluted to 10 cells/ml with a limiting dilution assay. The diluted cell suspension was distributed at 100 μl per well in a 96-well plate (Corning Life Sciences, Acton, MA). Wells containing no cells or more than a single cell were excluded from our study. The wells with a single cell were marked and inspected every day using an inverted microscope to count the number of cell clones. Fresh medium (25 μl) was added to each well every two days. After two weeks, the number of spheres in each marked well was evaluated [25].

### CD133 staining for flow cytometry

A sample of 1×10^7^ cells in suspension was collected, and the cells were resuspended in PBS with 0.5% BSA (BSA; Sigma-Aldrich) and 2 mM EDTA (Sigma-Aldrich). The cells were then incubated at 4 °C in the dark with cluster of differentiation 133/2 (CD133/2)- phycoerythrin antibodies (Miltenyi Biotec, Bergisch Gladbach, Germany) for 10 min. Mouse IgG2b antibodies (Miltenyi Biotec) were used for the isotype control group. The cells were washed with PBS, and the percentage of CD133^+^ cells was evaluated with flow cytometry (Beckman Coulter).

### Cytospin slide preparation and immunofluorescence

The cells were washed and resuspended in PBS at 5×10^5^/ml. A 100-μl aliquot of the cell suspension was centrifuged onto glass slides at 500 × *g* for 2 min by cytospin preparation. The cells were fixed in 4% paraformaldehyde (Sigma-Aldrich)/PBS for 10 min, rinsed with PBS, and permeabilized with 0.4% Triton X-100 (Sigma-Aldrich)/PBS for 30 min. After being blocked in 10% goat serum (Boster Biologic Technology, Wuhan, China) for 30 min, the cells were incubated with primary antibodies diluted in 10% goat serum overnight at 4 °C. The following primary antibodies were used: goat anti-Nanog (1:100 dilution; R&D Systems, Minneapolis, MN); goat anti-Oct3/4 (1:100 dilution; R&D Systems); mouse anti-Pax6 (1:100 dilution; Millipore); rabbit anti-Nestin (1:50 dilution; Boster Biologic Technology); goat antiglial fibrillary acidic protein (anti-GFAP; 1:50 dilution; Millipore); rabbit anti-Brn3b (1:100 dilution; Santa Cruz, Santa Cruz, CA); rabbit anti-Islet-1 (1:100 dilution; Santa Cruz), and goat anti-Rhodopsin (1:100 dilution; Millipore). The cells were subsequently incubated with fluorescein isothiocyanate– or Cy3-labeled secondary antibodies (R&D; Boster Biologic Technology) for 1 h at room temperature in the dark. After being washed three times with PBS for 5 min each, the samples were counterstained with 4',6-diamidino-2-phenylindole (Boster Biologic Technology). The negative controls were stained without the primary antibodies. The confocal fluorescence images were acquired using a laser-scanning microscope (LSM, Carl Zeiss, Thornwood, NY).

### Real-time polymerase chain reaction analysis of gene expression

The total RNA was extracted using TRI reagent (Ambion, Austin, TX), and the RNA was then treated with DNase I (Sigma-Aldrich) to remove any genomic DNA contamination. First-strand cDNA was synthesized with TaqMan reverse transcription reagents (Applied Biosystems, Darmstadt, Germany). The primers used for the real-time PCR are listed in Table 1. The quantitative PCR was performed with SYBR Green Master Mix Real-Time Core Reagents (Applied Biosystems) using an ABI 7500 (Applied Biosystems) according to the manufacturer's instructions. Human glyceraldehyde 3-phosphate dehydrogenase (*GAPD*) was used as the housekeeping gene for the amplifications, and the mRNA expression level of each gene relative to the housekeeping gene was calculated by the comparative threshold cycle method as previously described [26].

**Table 1:** Primer sequences used in real-time PCR.

**Gene name**	**Primer sequences**
CD133-F	GCACTCTATACCAAAGCGTCA
CD133-R	CCATACTTCTTAGTTTCCTCA
Oct3/4-F	CTTGAATCCCGAATGGAAAGGG
Oct3/4-R	CCTTCCCAAATAGAACCCCCA
Nanog-F	CCCTCCTCCCATCCCTCATA
Nanog-R	CAACCATACTCCACCCTCCA
Musashi-1-F	AGAGTGAGGACATCGTGGAGA
Musashi-1-R	GGCGTAGGTTGTGGCTTGGA
Nestin-F	GAGCAGCACTCTTAACTTACGA
Nestin-R	TTCCTACAGCCTCCATTCTTG
Rx-F	AGCATCAACTGGCTACTGTCTG
Rx-R	CTTATTCCATCTTTCCCACCT
Lhx2-F	CCTCACAAACGACTCTTACCAA
Lhx2-R	TGTTTCCAGGCGAGATCCTA
Chx10-F	CTCGTGATATGCTGCTTGTG
Chx10-R	GCCTGTGGCTTCGTAGATG
Pax6-F	TTCAGCACCAGTGTCTACCA
Pax6-R	TCATAACTCCGCCCATTCA
GAPDH-F	AGAAAAACCTGCCAAATATGATGAC
GAPDH-R	TGGGTGTCGCTGTTGAAGTC

### Dickkopf-related protein 1 and Lefty-A induced differentiation in retinal neurons

To differentiate the SLCCs into retinal neurons, embryonic stem cell (ES) [16-18] and iPSC [19] differentiation protocols were followed with some modifications. The SLCC colonies were dissociated into single cells by treatment with 0.15% trypsin (Sigma-Aldrich) and 0.02% EDTA for 5 min, followed by gentle trituration. The single-cell suspension was then replated in poly D-lysine (PDL)/laminin (Sigma-Aldrich)-coated six-well plates at a density of 1×10^5^ cells/ml. The differentiation medium contained DMEM/F12 (Gibco), B-27 Supplement (Gibco), N-2 Supplement (Gibco), 15% FBS, 0.1 mM nonessential amino acids (Gibco), 0.1 mM 2-mercaptoethanol (Gibco), 10 ng/ml bFGF, supplemented with 100 ng/ml Dickkopf-related protein 1 (Dkk-1; R&D Systems) and 500 ng/ml Lefty-A (R&D Systems), and applied for 10 days. The differentiation medium was then replaced with neuronal medium containing Neurobasal (Gibco), B-27 Supplement, N-2 Supplement, 5% FBS, 0.1 mM nonessential amino acids, and 2 mM L-glutamine (Gibco) for 10 days. The recombinant proteins were supplemented to continue the differentiation.

### Statistical analysis

At least three replicates were performed for each experiment. The data are presented as the mean±standard deviation (SD). Significance was evaluated using an appropriate Student *t* test for the comparison between two variables using Statistical Package for Social Sciences (SPSS) 13.0 (IBM, Armonk, NY), with a p value less than 0.05 taken as a statistically significant difference.

## Results

### Side population cells detected in the WERI-Rb1 cell line

Several tumors and tumor cell lines maintain a population of cells with characteristics of stem-like SP cells [27,28]. To determine whether SP cells exist in the human WERI-Rb1 line, we stained the cells with the fluorescent dye, Hoechst 33,342, and analyzed the cells with flow cytometry. After the dead cells and cellular debris were excluded based on the scatter signals, the WERI-Rb1 cells showed a small population of cells (0.075±0.017%) with SP cell characteristics (n=3). As seen in Figure 1A, a small but persistent number of cells displayed Hoechst exclusion. The addition of 50 μM verapamil (a multidrug resistance transporter 1 [MDR1] inhibitor) converted these Hoechst-negative cells into Hoechst-positive cells (Figure 1B), confirming that these cells were indeed SP cells.

### Stem-like cancer cells are highly clonogenic in vitro

**Figure 2:**
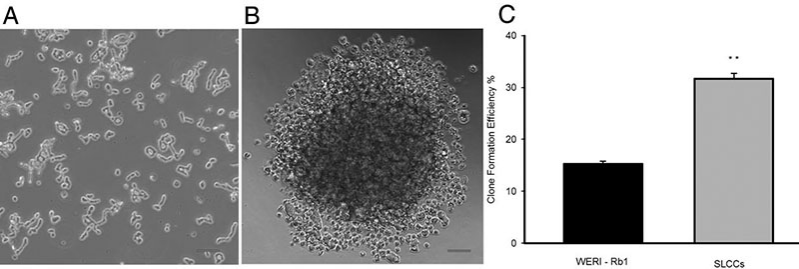
In vitro comparison of the clonogenic potential of stem-like cancer cells and WERI-Rb1 cells. **A**: The WERI-Rb1 cells grew in loose grape-like clusters when cultured in serum-RPMI-1640 medium. Scale bar=50 μm. **B**: Stem-like cancer cells (SLCCs) were plated as single-cell suspensions in 96-well plates. The expanded clones are displayed after 14 days. Scale bar=50 μm. **C**: The clone formation efficiency of WERI-Rb1 cells and SLCCs after 14 days was 15.26±0.93% and 31.70±1.89%, respectively (**p<0.01). The graph shows the mean±SD (n=3).

The formation of spherical colonies has been reported to be a property characteristic of stem/progenitor cells and verifies a high developmental and proliferative potency [29]. While maintained in a suspension of RPMI-1640 medium with 10% FBS, the WERI-Rb1 cells characteristically grew in loose grape-like clusters (Figure 2A). Serum-free medium was used to isolate and maintain the stem-like cancer cells. We found the SFM maintained an undifferentiated stem cell state and adding bFGF and EGF induced the proliferation of multipotent, self-renewing retinoblastoma stem cells. After 14 days of single-cell suspension culture in 96-well plates, the SLCCs displayed expanded clones (Figure 2B). As shown in Figure 2C, the rate of clone formation in the SLCCs was 31.70±1.89%, and the rate in the WERI-Rb1 cells was 15.26±0.93% (p<0.01, n=3).

### Increased number of CD133-positive cells in stem-like cancer cells

**Figure 3:**
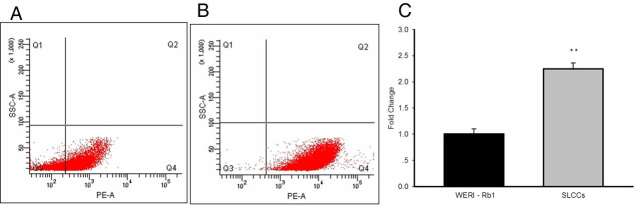
Higher expression levels of CD133 protein and genes in stem-like cancer cells. **A**-**B**: The percentage of CD133^+^ cells was determined with flow cytometric analysis using a phycoerythrin-conjugated anti-CD133 antibody. CD133^+^ cells (in the Q4 area) comprised 69.77±1.39% of the total cell population in the WERI-Rb1 cell line (Figure A), whereas CD133^+^ cells comprised 99.03±0.15% in the stem-like cancer cells (SLCCs; **B**:; p<0.001 with the Student *t* test). **C**: Real-time PCR quantification of the *CD133* gene showed 2.25±0.19-fold higher expression in the SLCCs (**p<0.01 with the Student *t* test). The graph shows the mean±SD (n=3).

CD133 has been found to be expressed on cancer stem cells [30,31], circulating endothelial progenitor cells [32,33], fetal neural stem cells [34,35], and other tissue-specific stem cells [36-38]. When stained with phycoerythrin-conjugated CD133 antibodies, the SLCCs contained more CD133^+^ cells than the WERI-Rb1 cells (99.03±0.15% versus 69.77±1.39%, p<0.001, n=3; Figure 3A,B). To quantify and compare the gene expression, real-time PCR was performed. A higher level of CD133 gene expression was found in the SLCCs (Figure 3C).

### Expression of embryonic stem and retinal stem cell markers in stem-like cancer cells

**Figure 4:**
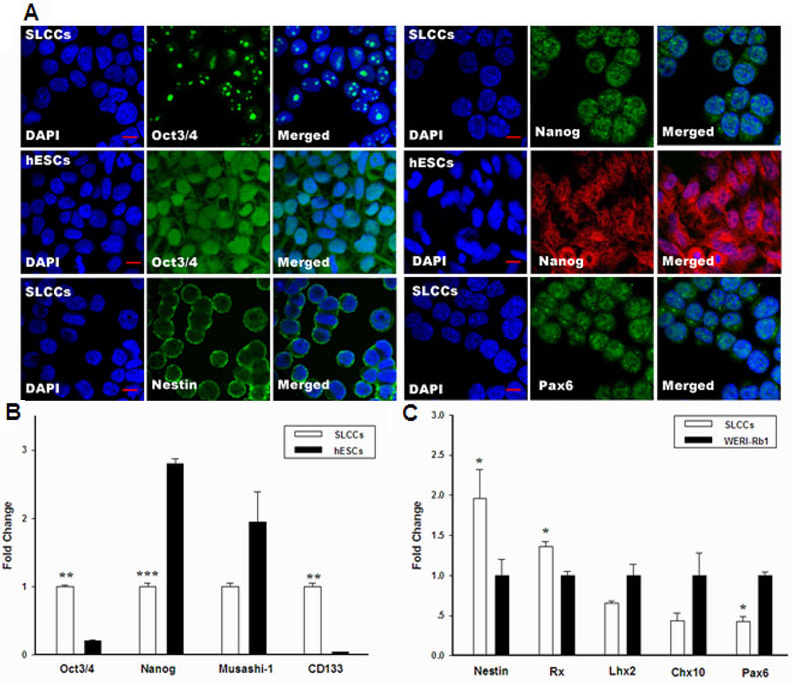
Embryonic stem and retinal stem cell marker expression in stem-like cancer cells. **A**: Immunofluorescence confirmed that the stem-like cancer cells (SLCCs) and human embryonic stem cells (hESCs) expressed embryonic stem cell markers, such as *Oct3/4* and Nanog, and the SLCCs expressed retinal stem cell markers, such as Nestin and *Pax6*. The nuclei were counterstained with 4',6-diamidino-2-phenylindole (blue). Scale bars=10 μm. **B**: Comparison of stem cell markers in hESCs and SLCCs with real-time PCR analysis. Nanog was expressed at a higher level in the hESCs (black bars) than in the SLCCs (white bars; ***p<0.001 with the Student *t* test), whereas *Oct3/4* and *CD133* were expressed at higher levels in the SLCCs (** p<0.01 with the Student *t* test). **C**: Real-time PCR quantification of retinal stem cell markers in the WERI-Rb1 line and SLCCs. Nestin and *Rx* were expressed at higher levels in the SLCCs (white bars; * p<0.05 with the Student *t* test), whereas *Pax6* was expressed at a higher level in the WERI-Rb1 cells (black bars; *p<0.05 with the Student *t* test). The graphs show the mean±SD (n=3).

Human octamer-binding transcription factor 3/4 (Oct 3/4) and Nanog are markers for pluripotency and self-renewal in embryonic stem cells [39], and Nestin and paired box gene 6 (Pax6) are undifferentiated retinal stem cell markers [22]. To determine whether the SLCCs expressed these markers, the cells were stained with antibodies against Oct3/4, Nanog, Nestin, or Pax6. The immunofluorescence results showed that these markers were detected in the SLCCs (Figure 4A-D).

In addition, a real-time PCR analysis was performed on human embryonic stem cells (hESCs) and SLCCs to compare ES marker genes, including Nanog and *Oct3/4,* the neural stem cell gene Musashi-1, and the cancer stem cell gene *CD133*. We found *Oct3/4* and *CD133* were expressed 4.74±0.37-fold and 25.12±3.65-fold higher in the SLCCs, respectively, whereas Musashi-1 and Nanog were expressed 1.95±0.77-fold and 2.80±0.13-fold higher in the hESCs, respectively (n=3; Figure 4E).

Retinal stem cell markers were also quantified in the WERI-Rb1 cells and SLCCs. Nestin and Retina and anterior neural fold homobox (*Rx*) were expressed 1.04±0.37-fold and 1.00±0.08-fold higher in the SLCCs, respectively, whereas LIM homebox gene 2 (*Lhx2*), CEH10 homodomain-containing homolog (*Chx10*), and *Pax6* were 1.51±0.27-, 2.24±0.22- and 2.45±0.69-fold higher in the WERI-Rb1 cells, respectively (n=3; Figure 4F).

### Dickkopf-related protein 1 and Lefty-A induce stem-like cancer cells into retinal neurons and upregulate retinal stem cell marker expression

**Figure 5:**
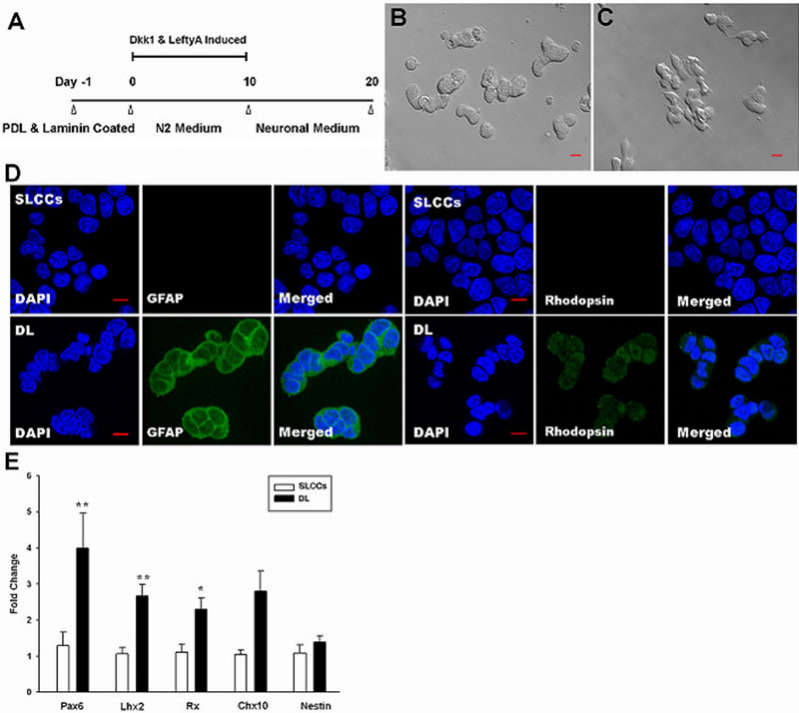
Stem-like cancer cell differentiation induced with Dickkopf-related protein 1 and Lefty-A (DL). **A**: Schematic diagram of the stem-like cancer cell (SLCCs) differentiation protocol. **B**: SLCCs plated on a poly-D-lysine/laminin-coated 6-well plate on day 0. **C**: Most of the SLCCs displayed neuron-like morphology with synapse-like structures after two to three days of induction. Scale bars=10 μm. **D**: An immunofluorescence assay showed that Dickkopf-related protein 1 (Dkk-1) and Lefty-A induced the neuron-like cell expression of glial fibrillary acidic protein (GFAP) and Rhodopsin (a photoreceptor marker) whereas SLCCs before differentiation with negative staining for GFAP and Rhodopsin. Scale bars=10 μm. **E**: Real-time PCR demonstrated that DL induction significantly upregulated the expression of *Pax6*, *Lhx2*, and *Rx*. The graphs show the mean±SD (n=3). White bars: control group. Black bars: DL-induced group. (*p<0.05, **p<0.01, ***p<0.001, with the Student *t* test).

To differentiate the SLCCs, previously described protocols were used with some modification (Figure 5A) [16-19]. In brief, the SLCCs grew adherently in N2 medium with Dkk-1 and Lefty-A for 10 days. Then the medium was changed into neuronal medium for the retinal neuron-like cells maintaining. The cells were replated on poly D-lysine/laminin-coated six-well plates (Figure 5B), and recombinant Dkk-1 (Wnt antagonist) and Lefty-A (Nodal antagonist) proteins were added to the N2 medium for differentiation. After two to three days of induction, most of the cells displayed neuron-like morphology (Figure 5C). The medium was then replaced with neuronal medium without Dkk-1 and Lefty-A on day 10.

We further confirmed the neuron-like cells with an immunofluorescence assay. The induced cells were positive for glial fibrillary acidic protein (GFAP; a marker of glial cells) and rhodopsin (a marker of photoreceptor cells; Figure 5D). However, expression of retinal ganglion cell markers, such as POU domain, class 4, transcription factor 2 (Brn3b) and ISL1 transcription factor (Islet-1), was not detected in this study.

To explore the mechanism of the Dkk-1- and Lefty-A-induced differentiation, real-time PCR was performed to analyze the retinal stem cell–related gene expression. We found significant upregulation of *Pax6*, *Lhx2*, and *Rx* after induction. The expression level was 3.99±2.97, 2.67±1.31, and 2.30±1.33 fold higher, respectively, whereas the expression level of *Chx10* and *Nestin* was 2.81±2.34 and 1.39±0.74 fold higher, respectively (n=3).

## Discussion

### Cancer stem cell characteristics in WERI-Rb1 cells

The WERI-Rb1 line, a retinoblastoma cell line, was established in 1974 from a one-year-old female Caucasian with no family history of retinoblastoma. The WERI-Rb1 line was found to be weakly tumorigenic compared with other Rb cell lines [20]. Since 2005, a small subpopulation of cells has been found to exhibit some characteristics of a cancer stem cell phenotype in retinoblastoma cell lines Y79, WERI-Rb27 [13], and in freshly isolated tumor cells [14,15]. The present study is the first that explores the cancer stem cell properties of the WERI-Rb1 line.

The major obstacle in CSC research is the difficulty in isolating them because of the lack of universally accepted markers for these cells. Use of the SP, a cell population enriched in stem cells, overcomes some of the barriers [10]. The SP is a distinct, small cell population composed of unstained cells in the left lower quadrant of a fluorescence-activated cell sorting profile. Goodell et al. demonstrated that the exclusion of Hoechst 33,342 by SP cells is an active process involving MDR1, a member of the adenosine-5′-triphosphate-binding cassette transporter family of transmembrane proteins [40]. Seigel et al. previously revealed that the ratio of the SP in the WERI-Rb27 line was 0.1% [13]. In the present study, we identified 0.075±0.017% SP cells in the WERI-Rb1 line. As the proportion of the SP in retinoblastoma is much smaller compared with other cancers, cell sorting and expansion of the SP are difficult to perform. Here, we used a serum-free medium to isolate and maintain the stem-like cancer cells according to described procedures [15,21,22]. Our data indicated the SLCCs had the ability to form expanded clones in the SFM and had a higher level of CD133 expression.

### Comparison of the stem cell markers between human embryonic stem cells and stem-like cancer cells

Oct3/4, a member of the POU (Pit-oct-unc) family of transcriptional factors, is well known as one of the four genes used to generate iPSCs [41]. As a pluripotency marker, Oct3/4 is thought to play a key role in maintaining pluripotency and the self-renewal of embryonic stem cells [42]. Interestingly, in this study, we provided the first evidence that human retinoblastoma stem cells expressed higher levels of *Oct3/4* than hESCs. This finding supplies the impetus for our further study of cancer stem cell differentiation.

A more recently described gene, Nanog, is thought to be a new “master gene” of ES cell pluripotency. Functionally, Nanog blocks differentiation; thus, negative regulation of Nanog is required to promote differentiation during embryonic development [43]. Compared with hESCs, the relatively reduced Nanog expression in retinoblastoma stem cells may indicate their higher differentiation properties.

Musashi-1 has been confirmed to be continuously expressed in neural stem cell–like cells [44-46], and it has been hypothesized that Musashi-1 plays a role in cell fate determination and differentiation through the Notch signaling pathway [47]. A previous study found Musashi-1 was expressed in retinoblastoma tumors and Y79 and WERI-Rb27 cell lines [12]. Together with our finding that Musashi-1 was expressed in the SLCCs of the WERI-Rb1 line, these results indicated neural stem characteristics in retinoblastoma.

### Stem-like cancer cells differentiation induced with Dickkopf-related protein 1 and Lefty-A

We confirmed the retinal stem cell genes Nestin and *Rx* were upregulated after culture of the WERI-Rb1 cells in serum-free medium. Therefore, we next asked whether the SLCCs maintain the differentiation potential of retinal stem cells that can differentiate into cell types of the mature retina under specific differentiation conditions [22,48]. Wingless (Wnt) and Nodal signals contribute to retinal neurogenesis during development [49,50], and blockade of endogenous Wnt and Nodal signals enhances and stabilizes the generation of neural cells in ES cell aggregates [51]. Our previous study also revealed that treating human iPS cells with Dkk-1 and Lefty-A, Wnt and Nodal antagonists, respectively, induced RPC marker expression and generated retinal neurons [19]. However, there is no report on the retinal neuron differentiation induced by Dkk-1 and Lefty-A in the SLCCs of Rb.

In this study, we found that the expression of retinal stem cell–related genes, such as Nestin, *Rx*, *Lhx2*, *Chx10*, and *Pax6*, was increased in Dkk-1- and Lefty-A-treated cell cultures. Among these genes, *Pax6*, *Lhx2*, and *Rx* were significantly upregulated. However, stimulated by Dkk-1 and Lefty-A, only *Pax6* was upregulated in iPSCs. These results suggested that the SLCCs in Rb were easier to differentiate into retinal neurons under Dkk-1 and Lefty-A induction conditions. We further confirmed that GFAP (a glial cell marker) and rhodopsin (a photoreceptor cell marker) were expressed in most of the cells after induction. However, the Dkk-1- and Lefty-A-treated culture failed in differentiating the SLCCs into Brn3b- and Islet-1-positive retinal ganglion cells (RGCs), suggesting that the inhibition of Wnt and Nodal signaling was insufficient for RGC differentiation.

In conclusion, our study revealed that the SLCCs of the WERI-Rb1 line expressed embryonic stem cell– and retinal stem cell–related genes and that they could be further induced into retinal neurons. This study used a strategy of a artificial change in cancer stem cell fate by transcription factors. Our findings may provide a new strategy for cell replacement therapy of irreversible retinal neurodegeneration diseases.
